# Performance of Bioelectrical Impedance and Anthropometric Predictive Equations for Estimation of Muscle Mass in Chronic Kidney Disease Patients

**DOI:** 10.3389/fnut.2021.683393

**Published:** 2021-05-21

**Authors:** Natália Tomborelli Bellafronte, Lorena Vega-Piris, Guillermina Barril Cuadrado, Paula Garcia Chiarello

**Affiliations:** ^1^Post-graduate Program in Health Sciences, Ribeirão Preto Faculty of Medicine, University of São Paulo, Ribeirão Preto, Brazil; ^2^Methodology Unit, Instituto de Investigación Sanitaria del Hospital Universitario de la Princesa, Madrid, Spain; ^3^Nephrology Department, Hospital Universitario de la Princesa, Madrid, Spain; ^4^Department of Health Sciences, Ribeirão Preto Faculty of Medicine, University of São Paulo, Ribeirão Preto, Brazil

**Keywords:** anthropometry, bioelectrical impedance, body composition, chronic kidney disease, dual energy X-ray absorptiometry, fat free mass, lean mass, sarcopenia

## Abstract

**Background:** Patients with chronic kidney disease (CKD) are vulnerable to loss of muscle mass due to several metabolic alterations derived from the uremic syndrome. Reference methods for body composition evaluation are usually unfeasible in clinical settings.

**Aims:** To evaluate the accuracy of predictive equations based on bioelectrical impedance analyses (BIA) and anthropometry parameters for estimating fat free mass (FFM) and appendicular FFM (AFFM), compared to dual energy X-ray absorptiometry (DXA), in CKD patients.

**Methods:** We performed a longitudinal study with patients in non-dialysis-dependent, hemodialysis, peritoneal dialysis and kidney transplant treatment. FFM and AFFM were evaluated by DXA, BIA (Sergi, Kyle, Janssen and MacDonald equations) and anthropometry (Hume, Lee, Tian, and Noori equations). Low muscle mass was diagnosed by DXA analysis. Intra-class correlation coefficient (ICC), Bland-Altman graphic and multiple regression analysis were used to evaluate equation accuracy, linear regression analysis to evaluate bias, and ROC curve analysis and kappa for reproducibility.

**Results:** In total sample and in each CKD group, the predictive equation with the best accuracy was AFFM_Sergi_ (men, *n* = 137: ICC = 0.91, 95% CI = 0.79–0.96, bias = 1.11 kg; women, *n* = 129: ICC = 0.94, 95% CI = 0.92–0.96, bias = −0.28 kg). AFFM_Sergi_ also presented the best performance for low muscle mass diagnosis (men, kappa = 0.68, AUC = 0.83; women, kappa = 0.65, AUC = 0.85). Bias between AFFM_Sergi_ and AFFM_DXA_ was mainly affected by total body water and fat mass. None of the predictive equations was able to accurately predict changes in AFFM and FFM, with all ICC lower than 0.5.

**Conclusion:** The predictive equation with the best performance to asses muscle mass in CKD patients was AFFM_Sergi_, including evaluation of low muscle mass diagnosis. However, assessment of changes in body composition was biased, mainly due to variations in fluid status together with adiposity, limiting its applicability for longitudinal evaluations.

## Introduction

Lean body mass reserve, whose major component is skeletal muscle, is an essential reserve that provides amino acids to support processes such as injury repair and the immune response (1, 2). Therefore, lean body mass plays an important role in clinical outcomes and disease progress, with low lean body mass related to worse prognosis and shorter survival (3). Appendicular lean mass, which encompasses the lean soft tissue in the limbs and is mainly composed of skeletal muscle mass, is the variable of choice for low muscle mass diagnosis (4) and is considered as a key parameter for nutritional status evaluation.

Body composition technologies such as computed tomography, magnetic resonance imaging and dual energy X-ray absorptiometry (DXA) provide objective information about skeletal muscle mass (5). DXA, the most available one of them, has become recognized for its ability to accurately and precisely measure total body composition (5), in a three compartment level (fat mass, lean soft tissue and bone mineral content) (6). However, DXA is not a bedside technique, requires patient transportation to the instrument and has high cost, thus hampering its use in routine practice.

Given the unfeasibility to apply reference methods in clinical settings, there is a growing interest in more suitable techniques for body composition evaluation, such as anthropometry and bioelectrical impedance analysis (BIA). Equations using BIA parameters have been validated to predict fat free mass (FFM, lean mass + bone mineral content) in healthy individuals (7). Methods for prediction of appendicular fat free mass (AFFM, FFM of the limbs) from BIA were also developed in healthy elderly subjects (8), in healthy adults with validation in heart, lung and liver transplant patients (9); and also in non-dialysis-dependent (NDD) chronic kidney disease (CKD) patients (10). Furthermore, methods to predict FFM have been developed from anthropometric measures in healthy (11) and non-obese adults (12), as well as in NDD (13) and hemodialysis (HD) (14) CKD patients.

Patients with CKD are vulnerable to loss of muscle mass due to several metabolic alterations derived from the uremic syndrome (15–18). The metabolic disorders already present in NDD patients (16) become more evident in more advanced stages of CKD when peritoneal dialysis (PD) and HD treatments are established (3). Even after kidney transplant (KTx) nutritional status is difficult to recover (18). Accordingly, body composition of CKD patients worsens with disease progression (19), with lean tissue loss, sometimes masked by edema and usually preceding weight loss. As body composition is a biomarker for prognosis and helps to monitor clinical interventions, its assessment needs to be part of CKD routine care, although it is often imprecise in clinical settings.

The routine use of simplified methods such as BIA and anthropometry in hospital settings may improve the evaluation of nutritional status allowing clinicians to identify individuals who would benefit most from targeted interventions, as well as to reliably monitor longitudinal changes. Therefore, the primary aim of this study was to assess the accuracy of equations using BIA and anthropometry measures, compared to DXA, to estimate FFM and AFFM for cross-sectional and longitudinal assessment, in NDD, HD, PD, and KTx CKD patients. Secondary, we evaluated the capacity of surrogate methods to diagnose low muscle mass.

## Materials and Methods

This was a longitudinal study that evaluated clinically stable NDD, HD, PD, and KTx CKD patients from a nephrology outpatient clinic at Ribeirão Preto Medical School University Hospital (University of São Paulo, São Paulo, Brazil) and at a specialized dialysis clinic, the Nephrology Service of Ribeirão Preto. Patients with CKD were enrolled between 2017 and 2019. Inclusion criteria were: age ≥18 and ≤60 years and under regular treatment for at least 6 months; for NDD patients, estimated glomerular filtration rate (eGFR) ≤44 ml/min; for PD patients, absence of peritonitis in the previous 30 days and dialysis for at least 3 months; for HD patients, 4-h dialysis session, three times per week, through an arteriovenous fistula and dialysis for at least 3 months; for KTx patients, transplant for at least 6 months and eGFR ≥ 45 ml/min.

Exclusion criteria were presence of malignant diseases, acute infections and inflammation, human immunodeficiency virus, chronic lung disease, liver and heart failure, pregnancy or lactation, having amputations or an electronic implant, wheelchair user or inpatient, body weight >140 kg or BMI >40kg/m^2^.

This study was conducted according to the principles of the Declaration of Helsinki, the protocol was approved by the human research ethics committee of Ribeirão Preto Medical School University Hospital. All selected patients were invited by the researcher and those interested in participating the study read and signed the informed consent form before the procedures began.

### Protocol and Data Collection

Demographic and clinical data were collected from electronic medical records. Blood samples were taken within 1 d to 1 week before study assessment to determine laboratory parameters. Analyses were performed at the University Hospital's central laboratory. Serum creatinine was determined by kinetic method (creatinine calibrated to IDMS: COBAS 6000 [Roche/Hitachi]). The eGFR was calculated by the Chronic Kidney Disease Epidemiology Collaboration equation (CKD-EPI Creatinine 2009 equation). Dialysis patients had the weekly clearance of urea adjusted for total body water, with Kt/V determination in the week before the assessment.

Anthropometry and BIA were assessed by a single experienced dietician and DXA by a trained technician. All measures were performed consecutively at the same visit, after an 8-h fast (for HD group, 4-h fast), in patients with empty urinary bladder, drainage of the peritoneal dialysate, 30 min after the midweek HD session, advised to avoid strenuous physical activity in the previous day, wearing light clothes, without shoes and on the right side of the body (except if a fistula was present).

The second evaluation was performed 10 ± 2 months later. All measurements were performed exactly with the same protocol as the first evaluation and with the same equipment, dietician and technician for DXA, BIA and anthropometry assessments.

### Anthropometric Measurements

Anthropometric measurements included body weight at the nearest 0.1 kg using a balance beam scale (Filizola^®^, São Paulo, Brazil); height, waist, calf and mid arm muscle circumferences at the nearest 0.1 cm. BMI was also calculated.

A flexible plastic tape with graduated scale was applied to measure circumferences. Waist circumference was measured at the umbilical scar level (20). Calf circumference was measured with subjects seating down, knees at 90° and at the calf greatest circumference (21). Mid arm circumference and triceps skinfold thickness were measured for mid arm muscle circumference calculation (22). Triceps skin fold thickness was assessed with an adipometer (Lange^®^, Cambridge Scientific Industries, Inc), three measures were taken and their mean was applied.

Hand grip strength was evaluated by a handheld pneumatic dynamometer (Charder^®^, MG 4800) with subjects seated and asked to grip as hard as possible for three times with 1 min intervals. The highest value was recorded (23).

### BIA

Multi-frequency spectroscopy BIA (BCM, Fresenius Medical Care, Bad Homburg, Germany) was performed with a tetra-polar whole-body wrist-to-ankle protocol (24) after 10-min adaptation in a supine position. The variables resistance, reactance, resistance index (resistance [ohm] divided by squared height [cm^2^]) and phase angle were provided at 50 kHz frequency; total body water, extracellular water and body cell mass (25), were provided by the software manufacturer.

Hydration status was assessed by over-hydration (OH) index provided by the BIA equipment (26, 27). Hydration disturbance was considered if OH > +1.1 L or OH < −1.1 L (28).

### DXA

DXA (Hologic Discovery A, USA, Bedford, MA) was performed to evaluate lean mass, appendicular lean mass, AFFM, FFM, fat mass and fat mass of the trunk (6). Daily calibration of the device was performed before each assessment by scanning a spine phantom. A whole body scan was done after 10 min adaptation in supine position.

### Predictive Equations of the Study

Supplementary Table 1 presents all the predictive equations herein evaluated, with information about BIA equipment applied, reference method against which the equation was validated and the population used for development of each equation. All BIA equations (7–10) evaluated in this study were developed with a tetra-polar whole-body wrist to ankle protocol.

### Low Muscle Mass Definition

As no cutoff points to define low muscle mass are available for CKD patients, we applied definition based on the literature: appendicular lean mass <15 kg for women and <20 kg for men, assessed by DXA, according to the new European consensus on sarcopenia (4).

### Statistical Analysis

Sample size was calculated based on the measurement of low muscle mass prevalence in CKD population. We assumed a minimum expected prevalence of 30% for all groups, with estimation of at least 80 patients for each CKD treatment group with a 5% precision and no correction for small population size (29).

Qualitative variables were presented as relative (percentage, %) and absolute (number, n) frequencies. Quantitative variables were expressed as measures of central tendency (mean) and dispersion (standard deviation).

Normality was tested with Shapiro-Wilk test with homoscedasticity evaluated. The differences between groups were performed by independent *T*-test or analysis of variance (ANOVA) adjusted by Bonferroni post-test, as appropriate. Categorical variables were compared by χ^2^-test.

For regression analysis, collinearity of data was observed.

Binary logistic regression analysis was performed to estimate the odds ratio for the potential diagnostic for low muscle mass. A multivariate analysis was carried out to evaluate de confounding effect of sex, age and weight.

DXA was considered the reference method for FFM and AFFM against which the predictive equations were validated for accuracy, for cross-sectional and body composition change data (prospective—cross-sectional data [Δ]).

Intraclass correlation coefficient (ICC) (30) was calculated to analyze agreement in a group level by comparing AFFM and FFM assessments by DXA analysis with predicted values.

The 5% error tolerance between measured and predict value (95% limits of agreement) was calculated as the percentage of the sample whose predicted value was within 0.95–1.05 fold the measured value.

Multiple regression analysis was also performed between measured and estimated values. The coefficient of determination (*R*^2^) is useful as it reflects the percentage of variation in the measurements by one method that is related to the variation in the other method (31). The standard error of the estimate provides information about the degree of the error (31).

Agreement at individual level between DXA data and predictive equations was evaluated by the Bland-Altman graphic (32) calculating the 95% limits of individual agreement (mean bias between the two methods ± 1.96 SD).

The best performing equation to be considered as surrogate for DXA analysis, in the total sample and in each CKD subgroup, was defined based on the combination of two criteria: (a) the highest ICC value with the narrowest 95% Confidence interval (95% CI); (b) the highest *R*^2^ and lowest standard error of the estimate. In addition, Bland-Altman bias with limit of agreement and also 5% error tolerance were taken into account.

For the best predictive equations, linear regression analyses were performed to determine the proportional bias between surrogate and reference methods.

Also, the inter-agreement between the DXA predictor of low muscle mass (appendicular lean mass) and the best predictive equation was quantified by Cohen‘s kappa coefficient. The sensitivity and specificity values were also estimated, and receiving operator characteristics curve analysis was carried out, assigning good reproducibility to area under the curve >80% (33).

Pearson correlation was applied to assess association between variables.

Significance was set at *p* ≤ 0.05 except if adjustment for multiple comparisons was necessary. All statistics were performed with SPSS version 23 (IBM, Armonk, NY, USA).

## Results

### Low Muscle Mass Diagnosis

CKD was secondary to systemic arterial hypertension (29%), glomerulonephritis (25%) and diabetes mellitus (10%). Clinical data, BIA, anthropometric and DXA measurements of CKD subgroups stratified by sex are shown in Table 1. PD patients were younger (*p* ≤ 0.008). Hand grip strength, appendicular lean mass and lean mass were lower for women than men. HD group had the lowest value of appendicular lean mass and fat mass, and NDD the highest measurement of appendicular lean mass. Total body water and OH were lower for women than men and for HD, and higher for NDD and PD patients. In the prospective analysis, all groups and both sexes lost muscle mass and strength, and gained fat mass.

**Table 1:** Clinical data, anthropometry, BIA and body composition analyze of CKD subgroups stratified by sex.

	**NDD**		**HD**		**PD**		**KTx**	
	**Men**	**Women**	**Men**	**Women**	**Men**	**Women**	**Men**	**Women**
**Cross-sectional data**								
Sample size	46	37	35	44	8	15	48	33
Age (years)	49 ± 10^a^	48 ± 10^a^	44 ± 12^a^	49 ± 8^a^	37 ± 12^b^	42 ± 12^b^	50 ± 8^a^	48 ± 9^a^
eGFR (mL/min/1.73 m^2^)	19.3 ± 9.27^a^	17.8 ± 7.53^a^	NA	NA	NA	NA	71.20 ± 16.78^b^	69.00 ± 20.79^b^
KT/V	NA	NA	1.48 ± 0.19	1.84 ± 0.72	2.72 ± 0.52	2.53 ± 0.51	NA	NA
Dialysis/KTx time (mo)	NA	NA	66 ± 55	81 ± 67	11 ± 9	18 ± 20	96 ± 64	86 ± 57
Weight (kg)	84 ± 15.6^a^	69 ± 16.3^a^^*^	69 ± 13.9^b^	64 ± 13^b^	79 ± 15^b^	59 ± 9^b^^*^	76 ± 12^b^	64 ± 11^b^^*^
Hand grip strength (kg)	38.8 ± 9.57^ab^	21.9 ± 5.77^ab^^*^	36.6 ± 7.21^a^	20.4 ± 4.98^a^^*^	45.5 ± 6.66^ab^	22.5 ± 6.85^ab^^*^	40.6 ± 7.55^b^	21.7 ± 4.90^b^^*^
Phase angle (°)	6.19 ± 1.06^a^	5.6 ± 0.77^a^^*^	6.34 ± 0.94^a^	5.4 ± 1.19^a^^*^	6.2 ± 0.65^a^	5.6 ± 1.01^a^	6.3 ± 0.78^a^	5.5 ± 0.68^a^^*^
Resistance index (cm^2^/ohm)	67.8 ± 10.73^a^	47.5 ± 9.43^a^	54.1 ± 11.8^b^	39.8 ± 8.05^b^	65.5 ± 10.14^ab^	44.0 ± 7.87^ab^	59.9 ± 8.66^a^	41.2 ± 6.09^a^
Total body water (L)	42.9 ± 5.46^a^	31.6 ± 4.93^a^^*^	36.2 ± 5.55^b^	27.7 ± 5.13^b^^*^	42.0 ± 6.08^abc^	29.5 ± 3.99^abc^^*^	39.0 ± 4.96^c^	28.1 ± 3.47^c^^*^
OH (L)	1.05 ± 2.01^a^	0.09 ± 1.38^a^^*^	−0.18 ± 1.99^b^	−0.66 ± 1.49^b^	1.30 ± 0.70^a^	0.25 ± 1.51^a^^*^	0.39 ± 1.02^ab^	−0.14 ± 0.87^ab^*^^
Body cell mass (kg)	28.7 ± 5.21^a^	18.6 ± 3.17^a^^*^	25.2 ± 4.50^b^	15.6 ± 4.69^b^^*^	28.6 ± 5.37^ab^	18.8 ± 3.22^ab^^*^	26.5 ± 5.28^ab^	15.8 ± 2.78^ab^^*^
Appendicular lean mass (kg)	23.5 ± 3.92^a^	15.9 ± 3.32^a^^*^	20.2 ± 3.54^b^	13.9 ± 2.78^b^^*^	23.9 ± 5.21^ab^	14.1 ± 2.30^ab^^*^	21.4 ± 3.27^ab^	13.8 ± 2.15^ab^^*^
Lean mass (kg)	50.9 ± 8.03^a^	36.5 ± 7.27^a^^*^	43.9 ± 7.75^b^	33.0 ± 5.89^b^^*^	48.1 ± 9.18^b^	31.7 ± 4.34^b^^*^	45.7 ± 6.49^b^	32.0 ± 5.05^b^^*^
Fat mass (kg)	24.5 ± 8.62^a^	26.1 ± 9.34^a^	17.3 ± 8.72^b^	24.2 ± 8.50^a^^*^	22.1 ± 7.92^a^	20.8 ± 5.63^a^	22.0 ± 6.67^a^	25.2 ± 7.30^a^^*^
**Δ** **data (prospective analysis—cross-sectional analysis)**								
Sample size	16	9	6	12	8	15	20	9
Weight (kg)	−1.35 ± 3.08	−0.21 ± 1.97	0.15 ± 4.64	−0.76 ± 1.76	−1.84 ± 2.78	−1.75 ± 3.35	0.18 ± 3.17	−2.72 ± 4.70
Hand grip strength (kg)	−3.71 ± 5.44	−0.70 ± 2.28	−2.00 ± 6.43	−1.07 ± 4.45	−0.70 ± 2.89	−1.08 ± 4.10	−2.24 ± 5.18	−0.65 ± 4.36
Phase angle (°)	−0.07 ± 0.64	−0.22 ± 0.46	−0.17 ± 0.89	−0.20 ± 0.51	0.45 ± 0.25	0.12 ± 0.62	0.00 ± 0.40	0.03 ± 0.36
Resistance index (cm^2^/ohm)	−3.42 ± 9.61	−0.27 ± 5.12	4.93 ± 14.18	1.01 ± 3.40	−4.42 ± 5.22	−2.78 ± 7.64	−0.72 ± 3.67	−1.90 ± 4.46
Total body water (L)	−1.68 ± 3.33	−0.34 ± 2.25	2.00 ± 4.98	0.05 ± 1.66	−1.42 ± 2.60	−1.34 ± 2.52	−0.19 ± 1.91	−1.00 ± 2.45
OH (L)	−0.32 ± 2.01	0.20 ± 0.82	0.80 ± 2.88	0.30 ± 0.82	−1.08 ± 0.47	−0.38 ± 1.53	−0.27 ± 0.86	−0.22 ± 0.46
Body cell mass (kg)	−1.70 ± 3.66	−0.66 ± 2.84	1.88 ± 5.22	−0.04 ± 2.51	−0.40 ± 4.22	−0.96 ± 1.68	−0.07 ± 2.44	−0.36 ± 2.78
Appendicular lean mass (kg)	−0.78 ± 1.78	−0.18 ± 1.18	−0.98 ± 1.95	−0.54 ± 1.32	−1.16 ± 3.02	−1.23 ± 1.51	−0.53 ± 0.98	−1.05 ± 2.27
Lean mass (kg)	−1.74 ± 3.61	−1.06 ± 2.83	−2.23 ± 3.81	−1.09 ± 2.83	−1.84 ± 4.45	−2.27 ± 2.59	−0.81 ± 2.27	−2.39 ± 5.63
Fat mass (kg)	0.18 ± 3.91	0.78 ± 2.51	2.26 ± 6.25	0.19 ± 2.68	0.25 ± 6.33	0.57 ± 2.74	1.05 ± 2.59	0.20 ± 4.70

*Data presented as mean ± standard deviation. BIA, bioelectrical impedance analyze; CKD, chronic kidney disease; HD, hemodialysis; KTx, kidney transplant; OH, over-hydration; NA, not applied; NDD, non-dialysis-dependent; PD, peritoneal dialysis. Body cell mass, resistance index, phase angle data by bioelectrical impedance analyze. Appendicular lean mass, lean mass, fat mass by dual energy X-ray absorptiometry analyze*.

* *Independent T-test between sex (p ≤ 0.05)*.

abc *ANOVA with Bonferroni post-test (p ≤ 0.008)*.

Low muscle mass affected more women (63%, *n* = 81) than men (37%, *n* = 51) (*p* ≤ 0.05), was more prevalent among HD (70%, *n* = 55) and less in NDD (30%, *n* = 24) than the other CKD patients (PD, 52%, *n* = 12; KTx, 52%, *n* = 42) (*p* ≤ 0.008).

The evaluation of factors related to the odds ratio for low muscle mass diagnosis is presented in Table 2. As NDD patients had the lowest prevalence of low muscle mass and the highest appendicular lean mass they were chosen as reference group. HD patients had more than 5 times and KTx about 3 times the risk for low muscle mass compared to NDD. Adiposity was a risk factor in the multivariate analysis adjusted for sex, age, and weight.

**Table 2:** Odds Ratio of low muscle mass diagnosis in total sample (*n* = 266).

	**Unadjusted**			**Adjusted by sex, age and weight**		
	**OR**	**95% CI**	***p***	**OR**	**95% CI**	***p***
Sex (reference: men)	0.351	0.214–0.578	0.000			
**CKD treatment (reference: NDD)**						
HD	5.978	3.032–11.788	0.000	5.155	1.779–14.940	0.003
PD	2.846	1.102–7.350	0.031	1.236	0.261–5.851	0.790
KTx	2.809	1.468–5.375	0.002	3.154	1.143–8.704	0.027
Weight (kg)	0.837	0.803–0.873	0.000			
BMI (kg/m^2^)	0.738	0.683–0.797	0.000	1.079	0.734–1.340	0.679
Hand grip strength (kg)	0.927	0.904–0.951	0.000	0.906	0.849–0.967	0.003
Phase angle (°)	0.649	0.501–0.840	0.0001	0.760	0.482–1.200	0.628
Resistance index (cm^2^/ohm)	0.863	0.832–0.894	0.000	0.831	0.772–0.895	0.000
Body cell mass (kg)	0.837	0.786–0.880	0.000	0.746	0.660–0.842	0.000
Fat mass (kg)	0.903	0.872–0.935	0.000	2.035	1.606–2.578	0.000
Fat mass of trunk (kg)	0.831	0.783–0.883	0.000	1.765	1.466–2.125	0.000

*BMI, body mass index; CKD, chronic kidney disease; HD, hemodialysis; KTx, kidney transplant; NDD, non-dialysis-dependent; OR, odds ratio; PD, peritoneal dialysis. Body cell mass, resistance index, phase angle data by bioelectrical impedance analyze. Fat mass and fat mass of the trunk by dual energy X-ray absorptiometry analyze. Low muscle mass according to the Revised European consensus (4) applying appendicular lean mass measurement by dual energy X-ray assessment. OR by binary logistic regression. For regression model analysis, the following variables were evaluated: age (years); estimated glomerular filtration ratio (mL/min/1.73 m^2^); KT/V; creatinine (mg/dL); urea (mg/dL); weight (kg), height (m); overhydration index (L); extracellular water (L); intracellular water (L); total body water (L); waist circumference (cm); calf circumference (cm); mid arm circumference (cm); mid arm muscle circumference (cm); resistance (ohm); reactance (ohm); the variables present in this table*.

### Accuracy of Predictive Equations to Estimate FFM and AFFM

**Figure 1:**
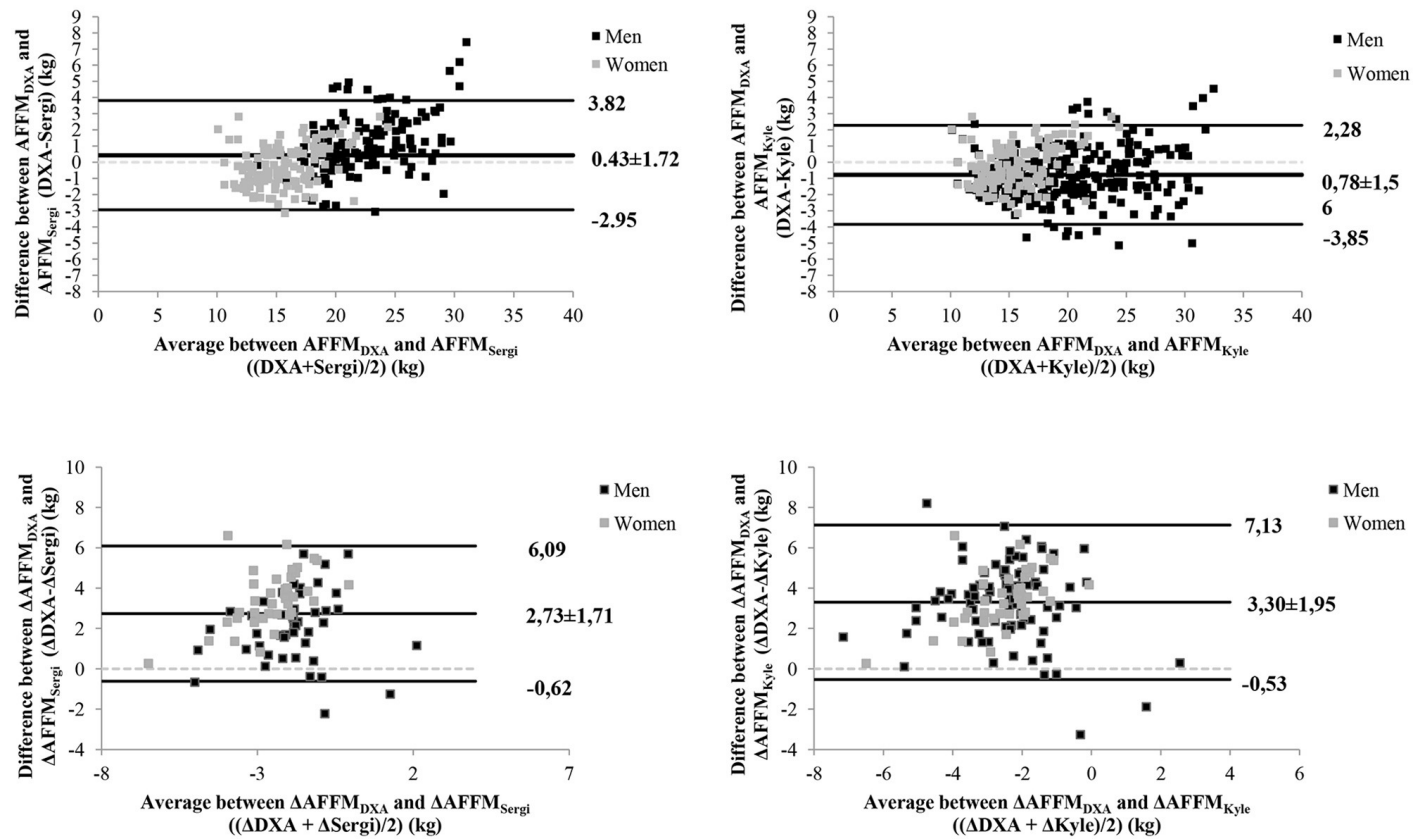
Bland-Altman plots for AFFM_Sergi_ and AFFM_Kyle_ predictive equations compared with DXA results, in total sample, for cross-sectional and body composition changes data (Δ).

The agreement of predicted AFFM and FFM in comparison with AFFM and FFM from DXA for the total sample is presented in Table 3, and for each CKD group as supplementary material (NDD, Supplementary Table 2; HD, Supplementary Table 3; KTx, Supplementary Table 4; it was not possible to evaluated the PD group because of its small sample size). Agreement analysis was also performed according to the results of *R*^2^ and standard error of the estimate in the total sample and is presented in Table 4 for cross-sectional and prospective evaluations. Bland-Altman and scatter plot graphics for AFFM_Sergi_ and AFFM_Kyle_ equations compared with AFFM by DXA are presented in Figure 1 and Supplementary Figure 1, respectively, for cross-sectional and body composition change data.

**Table 3:** Agreement between DXA and prediction equations for AFFM and FFM in total sample stratified by sex.

**Body composition variable**	**Men**												**Women**											
			**Bland-Altman** **analysis**				**ICC** **analysis**								**Bland-Altman** **analysis**				**ICC** **analysis**					
	**DXA or** **prediction** **equation**		**Bias** **(DXA-prediction)**		**LOA**		**ICC**	**(95% CI)**		**Pearson** **correlation**		**5%** **tolerance**	**DXA or** **prediction** **equation**		**Bias** **(DXA-prediction)**		**LOA**		**ICC**	**(95%CI)**		**Pearson** **correlation**		**5%** **tolerance**
	**x̄**	**SD**	**x̄**	**SD**	**Lower**	**Upper**	***r***	**Lower**	**Upper**	***r***	***p***	**% (n)**	**x̄**	**SD**	**x̄**	**SD**	**Lower**	**Upper**	***r***	**Lower**	**Upper**	***r***	***p***	**% (n)**
	**Cross-sectional data** ***n*** **=** **137**												**Cross-sectional data** ***n*** **=** **129**											
AFFM_DXA_ (kg)	23.16	4.11											15.32	3.02										
AFFM_Sergi_ (kg)	22.05	3.31	1.11	1.83	−2.47	4.70	0.915	0.795	0.956	0.90	0.000	50 (69)	15.61	2.60	−0.28	1.26	−2.76	2.19	0.945	0.921	0.962	0.91	0.000	42 (54)
AFFM_Kyle_ (kg)	23.90	3.76	−0.73	1.82	−4.30	2.83	0.935	0.892	0.959	0.90	0.000	44 (61)	16.16	2.93	−0.83	1.24	−3.28	1.61	0.935	0.815	0.969	0.91	0.000	39 (50)
AFFM_Macdonald_ (kg)	22.10	3.21	1.05	2.11	−3.08	5.18	0.804	0.686	0.880	0.86	0.00	42 (57)	13.86	2.53	1.45	1.82	−2.11	5.01	0.692	0.284	0.846	0.80	0.000	24 (31)
FFM_DXA_ (kg)	48.99	8.23											34.87	6.42										
FFM_TianHGS_ (kg)	50.32	6.65	−1.29	3.90	−8.93	6.35	0.852	0.782	0.898	0.88	0.000	42 (58)	36.25	5.90	−1.34	2.96	−7.14	4.46	0.865	0.768	0.917	0.89	0.000	41 (53)
FFM_TianMAMC_ (kg)	53.09	6.82	−4.10	3.77	−11.48	3.28	0.764	0.140	0.909	0.89	0.000	32 (43)	37.56	6.05	−2.69	3.00	−8.57	3.19	0.809	0.366	0.919	0.88	0.000	30 (39)
FFM_NooriHGS_ (kg)	40.22	7.85	8.80	8.69	−8.23	25.83	0.262	−0.048	0.505	0.42	0.000	22 (30)	23.72	5.12	11.18	6.54	−1.63	23.99	0.129	−0.076	0.360	0.38	0.000	5 (6)
FFM_NooriMAMC_ (kg)	48.32	6.12	0.66	4.04	−7.96	8.57	0.842	0.785	0.885	0.88	0.000	42 (57)	45.34	5.40	−10.46	3.00	−16.34	−4.58	0.342	−0.036	0.712	0.88	0.000	2 (3)
FFM_Hume_ (kg)	53.05	6.40	−4.06	4.13	−12.15	4.03	0.732	0.181	0.886	0.87	0.000	30 (41)	41.49	5.64	−6.61	3.16	−12.80	6.19	0.540	−0.078	0.833	0.87	0.000	7 (9)
FFM_Janssen_ (kg)	30.21	4.53	18.78	5.11	8.76	28.79	0.141	−0.033	0.437	0.83	0.000	0 (0)	18.91	3.44	15.96	4.11	7.90	24.01	0.118	−0.029	0.388	0.81	0.000	0 (0)
FFM_Lee_ (kg)	33.91	3.84	15.08	5.36	4.57	25.58	0.174	−0.052	0.486	0.85	0.000	0 (0)	23.37	3.39	11.50	3.91	3.83	19.16	0.203	−0.049	0.538	0.86	0.000	0 (0)
	**Δ** **data** ***n*** **=** **47**												**Δ** **data** ***n*** **=** **40**											
AFFM_DXA_ (kg)	−0.78	1.66											−0.78	1.58										
AFFM_Sergi_ (kg)	−2.86	1.50	2.09	1.73	−1.30	5.48	0.361	−0.194	0.671	0.41	0.000	0 (0)	−4.25	0.93	3.47	1.37	0.77	6.17	0.177	−0.115	0.504	0.50	0.000	0 (0)
AFFM_Kyle_ (kg)	−3.20	1.88	2.43	1.97	−1.43	6.29	0.339	−0.196	0.653	0.39	0.000	0 (0)	−5.08	1.16	2.43	1.97	−1.43	6.29	0.158	−0.085	0.480	0.52	0.000	0 (0)
AFFM_Macdonald_ (kg)	−0.31	1.84	−0.45	1.94	−4.25	3.35	0.385	0.117	0.603	0.39	0.000	0 (0)	−0.21	1.02	−0.56	1.43	−3.36	2.80	0.391	0.106	0.620	0.46	0.000	0 (0)
FFM_DXA_ (kg)	−1.48	3.19											−1.71	3.50										
FFM_TianHGS_ (kg)	−0.27	1.33	−1.18	2.95	−6.96	4.60	0.117	−0.144	0.378	0.17	0.263	0 (0)	−0.54	1.31	−1.17	3.00	−7.05	4.71	0.329	0.043	0.572	0.54	0.000	0 (0)
FFM_TianMAMC_ (kg)	−0.03	1.53	−1.47	3.16	−7.66	4.72	0.173	−0.081	0.419	0.25	0.09	0 (0)	−0.61	1.43	−1.09	3.03	−7.02	4.84	0.338	0.050	0.579	0.51	0.000	0 (0)
FFM_NooriHGS_ (kg)	−2.27	4.68	0.90	5.58	−10.03	11.83	−0.021	−0.311	0.274	−0.023	0.881	0 (0)	−0.80	3.49	−0.90	3.18	−7.13	5.33	0.472	0.324	0.747	0.58	0.000	0 (0)
FFM_NooriMAMC_ (kg)	0.04	1.33	−1.54	3.13	−7.67	4.59	0.155	−0.093	0.400	0.25	0.08	0 (0)	−0.51	1.23	−1.19	3.10	−7.26	4.88	0.279	−0.007	0.532	0.48	0.000	0 (0)
FFM_Hume_ (kg)	−0.18	1.08	−1.32	3.12	−7.43	4.79	0.123	−0.123	0.375	0.22	0.12	0 (0)	−0.39	0.96	−1.32	3.10	−7.39	4.75	0.243	−0.039	0.500	0.52	0.000	0 (0)
FFM_Janssen_ (kg)	−0.52	3.21	−0.93	3.63	−8.04	6.18	0.352	0.081	0.577	0.36	0.01	0 (0)	−0.35	2.14	−1.36	3.07	−7.37	4.65	0.403	0.115	0.631	0.49	0.000	0 (0)
FFM_Lee_ (kg)	−0.13	0.80	−1.36	3.10	−7.43	4.71	0.094	−0.152	0.347	0.22	0.12	0 (0)	−0.32	0.79	−1.38	3.16	−7.57	4.81	0.202	−0.075	0.464	0.53	0.000	0 (0)

*AFFM, appendicular fat free mass; DXA, dual energy X-ray absorptiometry; FFM, fat free mass; ICC, intraclass correlation coefficient; LOA, limits of individual agreement; Δ, body composition changes data (second assessment—first assessment). Bias calculated as DXA data—Prediction equation value; 5% tolerance between DXA and prediction equations (Prediction equation/DXA from 0.95 to ≤ 1.05)*.

**Table 4:** Performance of prediction equations in total sample (*n* = 266).

**Prediction equation**	***R***^**2**^ **(coefficient of determination)**	**Standard error of the estimate (kg)**
**Cross-sectional data** ***n*** **=** **266**		
AFFM_Sergi_	0.91	1.58
AFFM_Kyle_	0.91	1.57
AFFM_Macdonald_	0.86	1.98
FFM_TianHGS_	0.88	3.47
FFM_TianMAMC_	0.88	3.46
FFM_NooriHGS_	0.52	7.12
FFM_NooriMAMC_	0.63	6.27
FFM_Hume_	0.86	3.73
FFM_Janssen_	0.82	4.25
FFM_Lee_	0.84	4.05
**Δ** **data** ***n*** **=** **87**		
AFFM_Sergi_	0.14	1.50
AFFM_Kyle_	0.13	1.51
AFFM_Macdonald_	0.16	1.48
FFM_TianHGS_	0.13	2.97
FFM_TianMAMC_	0.14	3.09
FFM_NooriHGS_	0.05	3.11
FFM_NooriMAMC_	0.13	3.12
FFM_Hume_	0.13	3.11
FFM_Janssen_	0.16	3.06
FFM_Lee_	0.14	3.09

*AFFM, appendicular fat free mass; FFM, fat free mass*.

For cross-sectional data, considering the total sample and each CKD subgroup, FFM prediction equations did not performed well, AFFM_Sergi_ and AFFM_Kyle_ presented the best performance according to the highest ICC with narrowest 95%CI and the highest *R*^2^ with lowest standard error of the estimate, in addition to the lowest bias and limits of agreement as well as the highest percentage within the 5% tolerance. Regarding body composition changes, none of the predictive equations was able to accurately predict changes in AFFM and FFM, with all ICC lower than 0.5.

### Analysis of Interfering Factors for the Best Predictive Equations

We then investigated which variables were associated with bias between the best predictive equations (AFFM_Sergi_ and AFFM_Kyle_) and AFFM by DXA (Table 5). The differences between the two methods were affected by sex, resistance index, total body water, AFFM and fat mass for AFFM_Sergi_ (adjusted *r*^2^ = 0.95) and AFFM_Kyle_ (adjusted *r*^2^ = 0.94), for cross-sectional data and for body composition change data (AFFM_Sergi_ adjusted *r*^2^ = 0.96 and AFFM_Kyle_ adjusted *r*^2^ = 0.97).

**Table 5:** Simple and multiple linear regression models for factors associated with bias in agreement between DXA and prediction equations in total sample.

	**AFFM**_**DXA**_**—AFFM**_**Sergi**_ **(kg)**								**AFFM**_**DXA**_**—AFFM**_**Kyle**_ **(kg)**							
	**Simple linear regression model**				**Multiple linear regression model**				**Simple linear regression model**				**Multiple linear regression model**			
	**Coefficient**	**95% CI**		***p***	**Coefficient**	**95% CI**		***p***	**Coefficient**	**95% CI**		***p***	**Coefficient**	**95% CI**		***p***
		**Lower**	**Upper**			**Lower**	**Upper**			**Lower**	**Upper**			**Lower**	**Upper**	
**Cross-sectional data (*****n*** **=** **266)**																
Intercept					−0.443	−0.738	−0.148	0.003					0.775	0.491	1.060	0.000
Sex	1.400	1.018	1.783	0.000	−1.404	−1.559	−1.249	0.000	0.099	−0.280	0.478	0.607	−1.885	−2.035	−1.735	0.000
RI (cm^2^/ohm)	0.048	0.033	0.062	0.000	−0.038	−0.052	−0.024	0.000	−0.008	−0.022	0.006	0.263	−0.086	−0.099	−0.072	0.000
TBW (L)	0.096	1.018	1.783	0.000	−0.328	−0.363	−0.294	0.000	−0.002	−0.027	0.023	0.856	−0.322	−0.355	−0.288	0.000
AFFM (kg)	0.214	0.185	0.243	0.000	0.865	0.836	0.893	0.000	0.079	0.045	0.114	0.000	0.865	0.837	0.893	0.000
FM (kg)	−0.042	−0.067	−0.018	0.001	−0.077	−0.084	−0.070	0.000	−0.047	−0.069	−0.025	0.000	−0.074	−0.080	−0.068	0.000
**Body composition change (Δ) Data (*****n*** **=** **87)**																
Intercept					3.958	3.838	4.077	0.000					4.745	4.619	4.870	0.000
Sex	−1.384	−2.062	−0.705	0.000	−1.368	−1.522	−1.215	0.000	−1.874	−2.616	−1.131	0.000	−1.881	−2.042	−1.720	0.000
IR (cm^2^/ohm)	−0.073	−0.125	−0.022	0.006	−0.076	−0.107	−0.440	0.000	−0.121	−0.176	−0.065	0.000	−0.125	−0.158	−0.091	0.000
TBW (L)	−0.175	−0.305	−0.045	0.090	−0.224	−0.306	−0.142	0.000	−0.290	−0.431	−0.149	0.000	−0.222	−0.308	−0.136	0.000
AFFM (kg)	0.661	0.481	0.841	0.000	0.846	0.777	0.914	0.000	0.580	0.349	0.811	0.000	0.842	0.770	0.913	0.000
FM (kg)	−0.298	−0.378	−0.219	0.000	−0.083	−0.110	−0.550	0.000	−0.298	−0.396	−0.200	0.000	−0.086	−0.115	−0.057	0.000

*AFFM, appendicular fat free mass; DXA, dual energy X-ray absorptiometry; FM, fat mass; RI, resistance index; TBW, total body water. IR and TBW by bioelectrical impedance analysis. AFFM and FM by dual energy X-ray analysis. For regression model analysis, the following variables were evaluated: sex (1 man, 0 woman); age (years); estimated glomerular filtration ratio (mL/min/1.73 m^2^); KT/V; creatinine (mg/dL); urea (mg/dL); weight (kg), height (m); overhydration index (L); extracellular water (L); intracellular water (L); total body water (L); extracellular to total body water ratio; body cell mass (kg); lean mass, fat-free mass and fat mass by DXA analysis as well as their indexes; the variables present in this table*.

### Analysis of Reproducibility for the Best Predictive Equations

The reproducibility of AFFM_Sergi_ and AFFM_Kyle_ for low muscle mass diagnosis, using appendicular lean mass by DXA and according to the cutoffs proposed by the European revised consensus on sarcopenia (4), is presented in Table 6. The inter-agreement was quantified by kappa values, according to sex and CKD subgroups. In total sample and in each CKD subgroup, AFFM_Sergi_ had a better performance than AFFM_Kyle_, with Kappa from moderate to substantial agreement, and with good performance among NDD and KTx patients and poor performance among HD patients.

**Table 6:** Reproducibility of AFFM prediction equations to evaluate low muscle mass compared to DXA as reference.

	**Inter-agreement**		**Low muscle mass diagnostic performance**					
	**Kappa**	***p***	**Sensibility (%)**	**Specificity (%)**	**AUC**	**95% CI**		**Significance level**
						**Lower**	**Upper**	
**DXA** ***vs***. **AFFM**_**Sergi**_								
Total sample (*n =* 266)	0.676	0.000	73.48	94.02	0.838	0.786	0.889	0.000
Men (*n =* 137)	0.680	0.000	74.50	91.86	0.832	0.753	0.911	0.000
Women (*n =* 129)	0.650	0.000	72.83	97.91	0.854	0.788	0.920	0.000
NDD-CKD patients (*n =* 83)	0.809	0.000	78.26	98.33	0.883	0.780	0.986	0.000
Men (*n =* 46)	0.862	0.000	88.88	97.29	0.931	0.806	1.000	0.000
Women (*n =* 37)	0.757	0.000	71.42	100.00	0.857	0.708	1.000	0.000
HD-CKD patients (*n =* 79)	0.464	0.000	72.72	79.16	0.759	0.643	0.876	0.000
Men (*n =* 35)	0.364	0.031	30.00	66.66	0.683	0.501	0.866	0.050
Women (*n =* 44)	0.542	0.000	74.28	100.00	0.871	0.769	0.973	0.001
KTx-CKD patients (*n =* 81)	0.633	0.000	69.04	94.87	0.820	0.723	0.916	0.000
Men (*n =* 48)	0.689	0.000	70.00	96.42	0.832	0.701	0.964	0.000
Women (*n =* 33)	0.520	0.001	68.18	90.90	0.795	0.636	0.955	0.006
**DXA** ***vs***. **AFFM**_**Kely**_								
Total sample (*n =* 266)	0.510	0.000	53.03	97.76	0.754	0.694	0.814	0.000
Men (*n =* 137)	0.585	0.000	33.33	96.51	0.649	0.549	0.750	0.004
Women (*n =* 129)	0.341	0.000	65.43	100.00	0.827	0.757	0.897	0.000
NDD-CKD patients (*n =* 83)	0.570	0.000	47.82	100.00	0.739	0.600	0.878	0.001
Men (*n =* 46)	0.315	0.003	22.00	100.00	0.611	0.383	0.840	0.306
Women (*n =* 37)	0.691	0.000	64.28	100.00	0.821	0.659	0.984	0.001
HD-CKD patients (*n =* 79)	0.388	0.000	60.00	87.5	0.738	0.623	0.852	0.001
Men (*n =* 35)	0.283	0.069	50.00	80.00	0.650	0.466	0.834	0.134
Women (*n =* 44)	0.439	0.000	65.71	100.00	0.829	0.710	0.947	0.003
KTx-CKD patients (*n =* 81)	0.443	0.000	45.23	100.00	0.726	0.615	0.838	0.000
Men (*n =* 48)	0.280	0.005	25.00	100.00	0.625	0.458	0.792	0.143
Women (*n =* 33)	0.538	0.000	63.63	100.00	0.818	0.677	0.960	0.003

*AFFM, appendicular fat free mass; AUC, area under the curve; DXA, dual energy X-ray absorptiometry; HD, hemodialysis; KTx, kidney transplant therapy; NDD, non-dialysis-dependent. Patients were classified with low muscle mass according to cutoffs values for ALM proposed by revised European consensus (4): appendicular lean mass <15 kg for women and <20 kg for men, assessed by DXA*.

### Correlations

Given the poor performance of prediction equations in predict changes of body composition, we performed correlation analysis to investigate if there is some surrogate variable with good correlation with cross-sectional data and muscle mass changes, being as an alternative measurement for longitudinal evaluations (Table 7).

**Table 7:** Correlations between surrogate methods and DXA in total sample (*n* = 266).

**Variable**	**Correlation coefficient**	***p***
**Cross-sectional data** ***n*** **=** **266**		
**Correlations with appendicular lean mass (DXA)**		
Mid arm muscle circumference (cm)	0.73	0.000
Calf circumference (cm)	0.65	0.000
Resistance index (cm^2^/ohm)	0.91	0.000
Phase angle (°)	0.35	0.000
Body cell mass (kg)	0.82	0.000
AFFM_Sergi_ (kg)	0.95	0.000
AFFM_Kyle_ (kg)	0.95	0.000
**Correlation with len mass (DXA)**		
Mid arm muscle circumference (cm)	0.74	0.000
Calf circumference (cm)	0.65	0.000
Resistance index (cm^2^/ohm)	0.92	0.000
Phase angle (°)	0.29	0.000
Body cell mass (kg)	0.78	0.000
AFFM_Sergi_ (kg)	0.96	0.000
AFFM_Kyle_ (kg)	0.96	0.000
**Δ** **data** ***n*** **=** **87**		
**Correlation with** **Δ** **appendicular lean mass (DXA)**		
Mid arm muscle circumference (cm)		>0.05
Calf circumference (cm)	0.34	0.000
Resistance index (cm^2^/ohm)	0.68	0.000
Phase angle (°)		>0.05
Body cell mass (kg)	0.37	0.000
AFFM_Sergi_ (kg)	0.38	0.000
AFFM_Kyle_ (kg)	0.37	0.000
**Correlation with** **Δ** **lean mass (DXA)**		
Mid arm muscle circumference (cm)		>0.05
Calf circumference (cm)	0.34	0.000
Resistance index (cm^2^/ohm)	0.70	0.000
Phase angle (°)		>0.05
Body cell mass (kg)	0.40	0.000
AFFM_Sergi_ (kg)	0.41	0.000
AFFM_Kyle_ (kg)	0.40	0.000

*AFFM, appendicular fat free mass; Δ, body composiiton change (prospective assessment - cross-sectional assessment)*.

## Discussion

Our study showed that AFFM_Sergi_ was the best equation to predict AFFM in CKD, with good performance for low muscle mass diagnosis among NDD and KTx patients, and poor performance among HD patients. FFM equations did not perform well. Fat mass and total body water were important factors that interfered with accuracy of prediction equations, mainly for longitudinal evaluations. None of the predictive equations was accurate for assessment of changes in AFFM and FFM. Resistance index presented the best correlation with appendicular lean mass and lean mass in cross-sectional and longitudinal assessment, highlighting as an alternative measurement for longitudinal evaluations. Multivariate analysis after adjustment for sex, age and weight, revealed HD, KTx and adiposity as risk factors for low muscle mass.

AFFM_Sergi_ (8) equation was recommended by the revised European consensus on sarcopenia (4) as a way to standardize BIA estimation since its assessments vary widely depending on the device applied (34). AFFM_Kyle_ (9) was chosen because it was the first widely evaluated predictive equation for AFFM developed with spectroscopy BIA. Both equations were validated against DXA and presented the best performances. For the others equations, some factors may have interfered with the accuracy of the estimations, such as the protocol applied for body composition assessment. While Noori et al. (14) carried out measurements in a non-HD day, in our study evaluations were conducted after the midweek HD session. Probably, the different time-to-perform lower the accuracy, since fluid status is one of the main factors interfering with accuracy of BIA for body composition evaluations (35).

Macdonald et al. (10), developed the equation with NDD CKD patients with eGFR of 45.9 ± 28.8 ml/min, much higher than eGFR in our NDD group and lower than eGFR in our KTx sample. As AFFM_Macdonald_ is strongly associated with eGFR (10), it is important to take into consideration this parameter for AFFM_Macdonald_ estimations, and probably was the reason for the worse performance in our sample. Tian et al. (13) developed their equations from NDD CKD patients with an eGFR of 27 mL/min/1.73 m^2^. Although this value is higher than the eGFR in our sample, it is closer than eGFR from Macdonald sample. Tian equations presented better performance for FFM than AFFM_Macdonald_, although lower than AFFM_Sergi_ and AFFM_Kyle_.

Another important factor is the BIA equipment applied for the development and validation of the predictive equation. All BIA devices involve application of a weak and alternating current through the body. However, they are not interchangeable. The number of frequencies (simple, multiple frequency or spectroscopy), the electronic circuitry, and the mathematical models (linear regression–derived population-specific equations or mathematical biophysical modeling by Cole model and Hanai's mixture theory) (36) applied for each device result in different predictions of water and body composition and also in raw values that are not interchangeable (35). As could be seen in Supplementary Table 1, different BIA was applied for development of the equations herein evaluated.

All methods for evaluation of body composition are indirect, requiring assumptions that may not hold true in illness (5). Failure to account for the precision error in the reference method applied to validate the estimated measurement may contribute to misinterpretation and scaling error (37). This could contribute to decrease the accuracy of AFFM_Janssen_ (7), FFM_Lee_ (12), and FFM_Hume_ (11) as they apply other reference methods than DXA.

It is essential to establish the ability of bedside methods to detect changes over time. In our study, none of the evaluated predictive equations had at least a good ICC for muscle mass changes, including AFFM_Sergi_ and AFFM_Kyle_. We took into account that the expected level of change in the body composition compartment was sufficiently high to be detected by the reference method, and confirmed that it was also detectable by the bedside method (24). Our total sample had a mean percentage of change [(second assessment –first assessment)/first assessment ^*^100)] in AFFM of −3.71 ± 8.32%, measured by DXA, −1.37 ± 6.33% by AFFM_Sergi_ and −1.46 ± 7.32% by AFFM_Kyle_. Similarly to DXA, AFFM_Sergi_, and AFFM_Kyle_ were able to detect changes in AFFM, although the direction of the change (gain or loss) and the quantity were poorly predicted, as confirmed by the low ICC and correlation and scatter plot graphics with fixed bias. Probably the fluid shift present in CKD and also the variation of adiposity lowered the accuracy of longitudinal assessment, as total body water and fat mass were important bias for prediction values. Therefore, AFFM_Sergi_ or AFFM_Kyle_ might not be appropriate for follow-up analysis.

The advantages of BIA compared with reference methods are mainly the portability of the device, ease of use, and its affordability. Its use to assess body composition has increased in daily practice, mainly due to validation studies. Close adherence to recommended measurement protocols (38) as well as a standardized time to perform, are important to minimize potential errors, mainly for longitudinal evaluation (38). However, estimation of body composition in CKD is prone to error because underlying assumptions such as hydration of FFM at 73%, stable distribution of extracellular to intracellular water, and predictability of body geometry are not met in this disease, especially in patients with altered hydration or excess adiposity (38). Prediction of AFFM seems to be less affected by the underlying considerations, but, as shown by our results, has limited accuracy in prospective assessment. The use of segmental BIA could improve prediction, despite studies showing controversial results (39, 40).

As pointed out by Mulasi et al. (41) it is unlikely that any algorithm can be relied upon for accurate whole-body estimates in patients with excess adiposity or altered fluid status, both conditions present in CKD. Excess adiposity (BMI ≥ 25 kg/m^2^) was present in 60% of our total sample and over-hydration (OH > + 1.1) in 40%. As BIA provides indirect estimates of body composition from the measurement of resistance of body tissues to an electric current, raw data might be an option to predicted values (42). In our sample, the only parameter with good correlation with longitudinal variation in lean mass and appendicular lean mass was the resistance index. In agreement with other authors (42) we suggest that raw measurements of BIA could provide better objective biomarkers of nutritional status than predicted values. This is an important issue for BIA applicability in clinical settings, mainly in CKD where it is already applied for fluid management. Another point that should not be underestimated is that, more often lately, clinicians have limited time to search the literature to identify the most suitable equation for the patient being evaluated; therefore, the only alternative is to directly apply the prediction provided by the device without knowing its appropriateness or interpreting the raw data.

Low muscle mass was present in more than 30% of patients in all CKD groups, highlighting that the nutritional status was compromised even among non-elderly patients. The NDD group was less affected by low muscle mass, although these patients also presented an important prevalence. A higher prevalence of low muscle mass was observed in HD patients, in agreement with other studies (43) suggesting that muscle wasting could progress as kidney function declines (19). Likewise, increased inflammation and metabolic acidosis promoted by the dialysis process accelerate protein degradation (44). Similarly to our results, other studies showed an important prevalence of low muscle mass among KTx patients (45), which could be related to previous dialysis therapy (90% of our patients underwent HD before KTx with a median duration of 40 months, 6–65) and with body composition deterioration after transplant (18), demonstrating the challenges of recovering nutritional status in CKD.

CKD patients lost muscle mass and gained fat mass, worsening their nutritional status, which is associated with worse quality of life and higher mortality (46). Thus, screening, prevention, and treatment of muscle loss should have high priority in CKD patients.

Total fat mass and trunk fat mass are risk factors for low muscle mass. Systemic and muscle oxidative stress, persistent inflammation, insulin resistance and hormonal alterations due to the presence of excess adipose tissue (47) lead to changes in muscle metabolism. These result in reduction of lean mass to fat mass ratio (48). Accordingly, the risk of skeletal muscle loss should also be considered in CKD patients with obesity, the most challenging patients for nutritional status evaluation as not only fluid status may impair their body composition assessment but also excess adiposity could mask loss of muscle mass. As provided by our results, fat mass was an important factor in bias for prediction of AFFM. So, obese patients could benefit most from use of raw BIA data for longitudinal evaluation of muscle mass.

The study's main limitations are the exclusion of patients over 60 years of age, the low sample size of the PD group and the short time of longitudinal assessment, which precludes analysis of exposure/outcome association and limits the evaluation of prediction equations for assessment of changes in body composition. Additionally, body thickness, hydration status, and diseases with water retention can affect DXA results (6). Therefore, in our DXA analysis of body composition, data from CKD patients with excess fat and fluid retention could be prone to errors that may promote a scaling error leading to misinterpretation of predicted values. On the other hand, the present study presents several strengths. First, the evaluation of CKD patients was carried out under treatment conditions such as HD and PD, as well as for NDD patients and patients after KTx therapy, which are more rarely evaluated. Moreover, the study includes DXA as reference method applied to all patients, together with a cross-sectional and longitudinal assessment. Additionally, we evaluated a wide variety of BIA and anthropometry predictive equations through a comprehensive statistical analysis including not only assessment of accuracy and agreement but also sensitivity and specificity analysis with discriminative power to detect low muscle mass.

In conclusion, accuracy and reproducibility analysis in the present study indicates that the predictive equation AFFM_Sergi_ can be an alternative technique to assess muscle mass in CKD patients, and could be useful for low muscle mass diagnosis in NDD and KTx patients, with limited applicability for patients under HD. However, prediction of changes in AFFM may be biased, mainly due to variations in fluid status together with the presence of high adiposity, limiting the applicability of AFFM_Sergi_ equation for longitudinal evaluations. As resistance index from BIA presented the best correlation with cross-sectional and longitudinal data of lean mass evaluated by reference method, we suggest that raw data from BIA could provide better information about progression of muscle mass in CKD, mainly in patients with dehydration or hiperhydration and excess adiposity. More studies are needed to better understand the relationship between nutritional status and also clinical prognosis with raw data from BIA as a way to easily obtain quick and objective measurements suitable to be tracked over time. Body composition evaluations are a priority for CKD patients, given the worsening of their nutrition status over time. Patients in HD, after KTx and with obesity are at higher risk for low muscle mass diagnosis.

## Data Availability Statement

The raw data supporting the conclusions of this article will be made available by the authors, without undue reservation.

## Ethics Statement

The studies involving human participants were reviewed and approved by Human Research Ethics Committee of Ribeirao Preto Medical School University Hospital. The patients/participants provided their written informed consent to participate in this study.

## Author Contributions

NB, PC, and GC contributed to the conception of the research. NB contributed to the design of the research, acquisition of the data, and drafted the manuscript. NB and LV-P contributed to the analysis and interpretation of data. All authors critically revised the manuscript, agree to be fully accountable for ensuring the integrity and accuracy of the work, and read and approved the final manuscript.

## Conflict of Interest

The authors declare that the research was conducted in the absence of any commercial or financial relationships that could be construed as a potential conflict of interest.

## Abbreviations

AFFM: appendicular fat free mass
ANOVA: analyses of variance
BIA: multifrequency bioelectrical impedance spectroscopy
BMI: body mass index
CKD: chronic kidney disease
DXA: dual energy X-ray absorptiometry
eGFR: estimated glomerular filtration rate
FFM: fat free mass
HD: hemodialysis
ICC: intraclass correlation coefficient
KTx: kidney transplant
NDD: non-dialysis-dependent
OH: over-hydration
PD: peritoneal dialysis
*R*^2^: coefficient of determination
Δ: body composition changes (prospective measurement—cross-sectional measurement)
95% CI: 95% confidence intervals.
