# Supplementary material for: Performance of Bioelectrical Impedance and Anthropometric Predictive Equations for Estimation of Muscle Mass in Chronic Kidney Disease Patients
Source: Front Nutr. 2021 May 21;8:683393. doi: 10.3389/fnut.2021.683393 (PMC8177428; doi:10.3389/fnut.2021.683393)
Supplement: Supplementary file 1 [file Image_1.pdf]

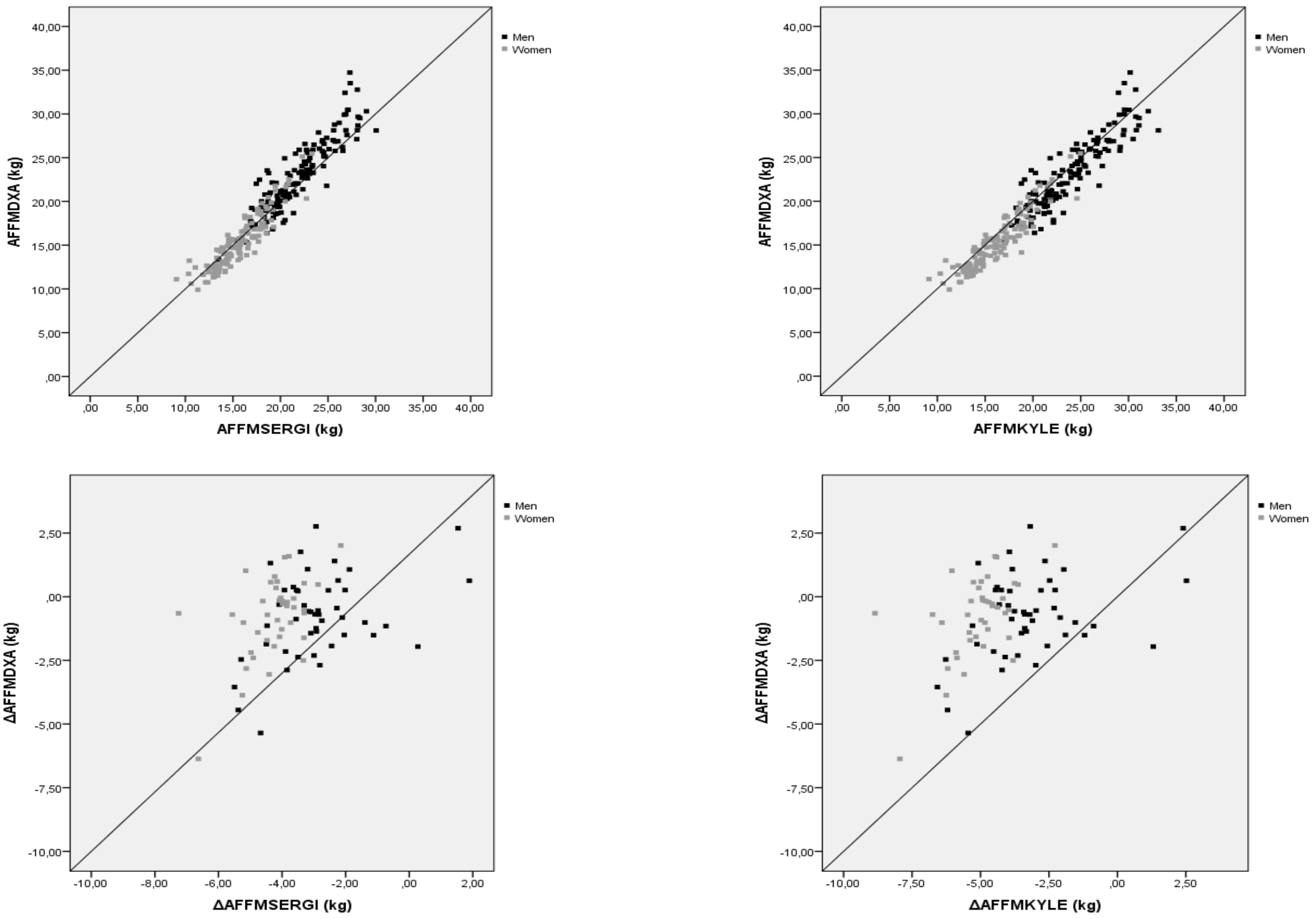

Fig. S1. Scater plots for $AFFM_{Sergi}$ and $AFFM_{Kyle}$ predictive equations compared with DXA results, in total sample for cross-sectional (total, n=266; men, n=137; women, n=129) and body composition changes (total, n=87; men, n=47; women, n=40) data.
