# Supplementary material for: Performance of Bioelectrical Impedance and Anthropometric Predictive Equations for Estimation of Muscle Mass in Chronic Kidney Disease Patients
Source: Front Nutr. 2021 May 21;8:683393. doi: 10.3389/fnut.2021.683393 (PMC8177428; doi:10.3389/fnut.2021.683393)
Supplement: Supplementary file 2 [file Table_1.pdf]

TABLE S1. AFFM and FFM by BIA and anthropometric predictive equations

Abbreviation	Equation	BIA equipment	Reference Method	Population	Ref.
**Predictive Equations developed with BIA data**					
AFFM$_{Kyle}$ (kg)	-4.211+ (1.909*sex) + (0.012*age) + (0.095*weight) + (0.267*IR) + (0.058*Xc) Sex, female = 0, male = 1; weight (kg); age (years). Data from BIA: IR (cm^2/ohm), Xc (ohm), both at 50 kHz frequency	BIS (Xitron 4000B, Xitron Technologies, Inc, San Diego, CA, USA)	DXA (QDR 4500A, Hologic Inc., Waltham, MA, USA)	24- to 94-year old healthy Caucasians	[9]
AFFM$_{Sergi}$ (kg)	-3.964 + (1.384*sex) + (0.095*weight) + (0.227*IR) + (0.064*Xc) Sex, female = 0, male = 1; weight (kg). Data from BIA: IR (cm^2/ohm), Xc (ohm), both at 50 kHz frequency	SFBIA (BIA 101 Anniversary AKERN/RJL Systems; Florence, Italy)	DXA (Hologic QDR Discovery A, Hologic Italy)	healthy elderly Caucasians (age > 60 years)	[8]
AFFM$_{Macdonald}$ (kg)	-11.626 – (2.092*sex) – (0.05*age) + (0.08553*height) + (0.292*IR) + (0.06983*Xc) Sex, female = 1, male = 0; age (years); height (cm). Data from BIA: IR (cm^2/ohm), Xc (ohm), both at 50 kHz frequency	BIS (Hydra ECF/ICF 4200, Xitron Technologies, San Diego, USA)	DXA (QDR1500, software version 5.72, Hologic, Waltham, USA).	NDD CKD patients	[10]
FFM$_{Janssen}$ (kg)	5.102 + (3.825*sex) – (0.071*age) + (0.401*IR) Sex, female = 0, male = 1; age (years). Data from BIA: IR (cm^2/ohm)	SFBIA (101B BIA analyzer, RJL Systems, Detroit, MI)	MRI (General Electric 1.5-T scanner, Milwaukee, WI)	Caucasian subjects (18–86 years)	[7]
**Predictive Equations developed with anthropometric data**					
FFM$_{Hume}$ (kg)	(0.32810*weight) + (0.33929*height) – 29.5336. Weight (kg); height (cm)	NA	antipyrine space method	subjects (37-80 years)	[11]
FFM$_{Lee}$ (kg)	(6.6*sex) – (0.098*age) + (0.244*Weight) + (7.80*height) + race - 3.3 Sex, male =1, female = 0; race, white or Hispanic = 0; age (years); weight (kg); height (m)	NA	MRI (1.5 Tesla scanners, 6X Horizon; General Electric, Milwaukee)	20- to 81-year old healthy subjects	[12]
FFM$_{TianHGS}$ (kg)	6.82*sex) + (0.18* height) + (0.40*weight) + (HGS* 0.01) - 18.12 Sex, male =1, female = 0; height (cm); weight (kg); HGS (kg)	NA	DXA (Orland Series XR-800 Pen Beam X-ray Bone Densitometer)	18- to 81-year old NDD CKD patients	[13]
FFM$_{TianMAMC}$ (kg)	(7.36*sex) + (0.22*height) + (0.37*weight) + (0.24*MAMC) – 26.34				

	Sex, male =1, female = 0; height (cm); weight (kg); MAMC (cm)				
$FFM_{NooriHGS}$ (kg)	(5.15*sex) + (0.33*weight) + (0.74*height) + (9.09*HGS) - 29.06<br>Sex, female = 1, male = 0; weight (kg); height (inches), HGS (kg)	NA	DXA(Hologic Series Delphi-A Fan Beam, software version 12.4, Hologic Inc)	HD CKD patients (mean age of 49±11 years) in non-HD days	[14]
$FFM_{NooriMAMC}$ (kg)	(5.52*sex) + (0.28*weight) + (0.82*height) + (0.28*MAMC) - 35.30<br>Sex, female = 1, male = 0; weight (kg); height (inches), MAMC (cm)				

AFFM, appendicular fat free mass; BIA, bioelectrical impedance analyze; BIS, multifrequency spectroscopy bioelectrical impedance analyze; CKD, chronic kidney disease; DXA, dual energy X-ray absorptiometry; FFM, fat free mass; HD, hemodialysis patients; HGS, hand grip strength; IR, impedance ratio; MAMC, mid arm muscle circumference; MRI, magnetic resonance image; NA, not applied; Ref, bibliographic reference; NDD, non-dialysis-dependent patients; SFBIA, single-frequency bioelectrical impedance analyze.
