# Supplementary material for: Performance of Bioelectrical Impedance and Anthropometric Predictive Equations for Estimation of Muscle Mass in Chronic Kidney Disease Patients
Source: Front Nutr. 2021 May 21;8:683393. doi: 10.3389/fnut.2021.683393 (PMC8177428; doi:10.3389/fnut.2021.683393)
Supplement: Supplementary file 3 [file Table_2.pdf]

TABLE S2. Agreement between DXA and prediction equations in non-dialysis-dependent sample stratified by sex

Body Composition Variable	Men												Women											
	DXA or Prediction Equation		Bland-Altman analysis				ICC analysis			Pearson correlation		5% Tolerance	DXA or Prediction Equation		Bland-Altman analysis				ICC analysis			Pearson correlation		5% Tolerance
			Bias (DXA-Prediction)		LOA		ICC	(95%CI)							Bias (DXA-Prediction)		LOA		ICC	(95%CI)				
	$\bar{x}$	SD	$\bar{x}$	SD	lower	upper	r	lower	upper	r	p	% (n)	$\bar{x}$	SD	$\bar{x}$	SD	lower	upper	r	lower	upper	r	p	% (n)
	Cross-sectional data n = 46												Cross-sectional data n = 37											
**AFFM$_{DXA}$ (kg)**	24.81	4.12											16.87	3.44										
**AFFM$_{Sergi}$ (kg)**	23.81	3.22	0.99	1.99	-2.91	4.89	0.828	0.654	0.910	0.88	0.00	52 (24)	16.93	2.78	-0.05	1.32	-2.63	2.53	0.913	0.837	0.954	0.93	0.00	38 (14)
**AFFM$_{Kyle}$ (kg)**	25.93	3.61	-1.12	2.02	-5.07	2.83	0.831	0.636	0.915	0.87	0.00	48 (22)	17.67	3.16	-0.79	1.30	-3.33	1.75	0.897	0.829	0.954	0.93	0.00	43 (16)
**AFFM$_{Macdonald}$ (kg)**	23.64	2.83	1.16	2.38	-3.05	5.82	0.738	0.500	0.837	0.83	0.00	39 (18)	15.13	12.11	1.73	2.25	-2.68	6.14	0.584	0.151	0.798	0.77	0.00	24 (9)
**FFM$_{DXA}$ (kg)**	52.87	8.31											37.90	7.47										
**FFM$_{TianHGS}$ (kg)**	53.18	6.72	-0.31	3.64	-7.44	6.82	0.806	0.602	0.875	0.84	0.00	39 (18)	38.40	6.65	-0.49	2.71	-5.80	4.82	0.880	0.804	0.930	0.93	0.00	43 (16)
**FFM$_{TianMAMC}$ (kg)**	56.08	6.79	-3.13	3.54	-10.06	3.80	0.825	0.382	0.932	0.91	0.00	39 (18)	39.69	6.69	-1.78	2.79	-7.24	3.68	0.878	0.701	0.890	0.93	0.00	40 (15)
**FFM$_{NooriHGS}$ (kg)**	39.99	8.90	12.88	9.27	-5.28	31.04	0.199	-0.093	0.489	0.42	0.00	9 (4)	24.34	5.33	13.55	8.25	-2.62	29.72	0.061	-0.066	0.241	0.20	0.28	5 (2)
**FFM$_{NooriMAMC}$ (kg)**	50.82	5.90	2.12	3.99	-5.70	9.94	0.801	0.600	0.870	0.81	0.00	43 (20)	47.21	5.51	-9.30	3.28	-6.42	-2.87	0.437	-0.052	0.793	0.91	0.00	5 (2)
**FFM$_{Hume}$ (kg)**	55.68	6.18	-2.80	3.97	-10.58	4.98	0.797	0.479	0.908	0.89	0.00	41 (19)	43.50	5.48	-5.60	3.51	-12.47	1.27	0.629	-0.085	0.876	0.89	0.00	1 (5)
**FFM$_{Janssen}$ (kg)**	32.67	4.03	20.30	5.60	9.32	31.27	0.110	-0.033	0.379	0.80	0.00	0 (0)	20.74	3.71	17.16	5.01	7.34	26.97	0.122	-0.037	0.413	0.80	0.00	0 (0)
**FFM$_{Lee}$ (kg)**	35.51	3.77	17.36	5.43	6.71	28.00	0.140	-0.042	0.444	0.86	0.00	0 (0)	24.59	3.75	13.31	4.25	4.98	21.64	0.210	-0.044	0.571	0.92	0.00	0 (0)

AFFM, appendicular fat free mass; DXA, dual energy X-ray absorptiometry; FFM, fat free mass; ICC, intraclass correlation coefficient; LOA, limits of individual agreement. Bias calculated as DXA data - Prediction equation value; 5% tolerance between DXA and prediction equations (Prediction equation/DXA from ≤0.95 to ≤1.05).
