# Supplementary material for: Performance of Bioelectrical Impedance and Anthropometric Predictive Equations for Estimation of Muscle Mass in Chronic Kidney Disease Patients
Source: Front Nutr. 2021 May 21;8:683393. doi: 10.3389/fnut.2021.683393 (PMC8177428; doi:10.3389/fnut.2021.683393)
Supplement: Supplementary file 4 [file Table_3.pdf]

TABLE S3. Agreement between DXA and prediction equations in hemodialysis sample stratified by sex

Body Composition Variable	Men												Women											
	DXA or Prediction Equation		Bland-Altman analysis				ICC analysis			Pearson correlation		5% Tolerance	DXA or Prediction Equation		Bland-Altman analysis				ICC analysis			Pearson correlation		5% Tolerance
			Bias (DXA-Prediction)		LOA		ICC	(95%CI)							Bias (DXA-Prediction)		LOA		ICC	(95%CI)				
	$\bar{x}$	SD	$\bar{x}$	SD	lower	upper	r	lower	upper	r	p	% (n)	$\bar{x}$	SD	$\bar{x}$	SD	lower	upper	r	lower	upper	r	p	% (n)
	Cross-sectional data n = 35												Cross-sectional data n = 44											
**$AFFM_{DXA}$ (kg)**	21.30	3.71											14.75	2.95										
**$AFFM_{Sergi}$ (kg)**	20.12	3.12	1.17	1.86	-2.47	4.81	0.809	0.636	0.914	0.87	0.00	51 (18)	14.99	2.73	-0.24	1.23	-2.65	2.17	0.904	0.832	0.946	0.91	0.00	50 (22)
**$AFFM_{Kyle}$ (kg)**	21.67	3.58	-0.36	1.86	-4.00	3.28	0.869	0.757	0.931	0.87	0.00	43 (15)	15.39	3.02	-0.64	1.15	-2.89	1.61	0.906	0.778	0.955	0.93	0.00	41 (18)
**$AFFM_{Macdonald}$ (kg)**	20.56	3.24	0.74	2.08	-3.33	4.81	0.808	0.623	0.900	0.83	0.00	43 (15)	13.09	3.00	1.65	1.51	-1.30	4.60	0.756	0.110	0.912	0.87	0.00	25 (11)
**FFM_{DXA} (kg)**	45.59	7.95											34.23	6.13										
**$FFM_{TianHGS}$ (kg)**	47.01	6.57	-1.38	4.35	-9.90	7.14	0.805	0.608	0.904	0.84	0.00	29 (10)	35.79	5.98	-1.55	3.05	-7.52	4.42	0.848	0.683	0.923	0.87	0.00	36 (16)
**$FFM_{TianMAMC}$ (kg)**	49.73	6.78	-4.03	4.20	-12.26	4.20	0.732	0.168	0.897	0.85	0.00	34 (12)	37.13	6.24	-2.90	3.10	-8.97	3.17	0.790	0.273	0.919	0.87	0.00	27 (12)
**$FFM_{NooriHGS}$ (kg)**	37.50	6.70	8.13	8.80	-9.11	25.37	0.187	-0.085	0.462	0.30	0.08	20 (7)	22.80	4.75	11.42	5.97	-0.28	23.12	0.129	-0.075	0.393	0.42	0.00	0 (0)
**$FFM_{NooriMAMC}$ (kg)**	45.42	6.30	0.17	4.22	-8.10	8.44	0.803	0.619	0.911	0.85	0.00	29 (10)	44.98	5.87	-10.74	2.60	-15.83	-5.64	0.348	-0.026	0.733	0.91	0.00	0 (0)
**FFM_{Hume} (kg)**	50.12	6.56	-4.53	4.37	-13.09	4.03	0.690	0.077	0.881	0.83	0.00	23 (8)	41.08	6.33	-6.85	2.52	-11.78	-1.91	0.573	-0.054	0.866	0.92	0.00	5 (3)
**$FFM_{Janssen}$ (kg)**	27.47	4.65	18.12	5.05	8.22	28.01	0.144	-0.034	0.463	0.80	0.00	0 (0)	17.62	3.34	16.61	3.40	9.94	23.27	0.115	-0.019	0.401	0.91	0.00	0 (0)
**FFM_{Lee} (kg)**	32.16	3.49	13.43	5.43	2.78	24.07	0.180	-0.063	0.514	0.82	0.00	0 (0)	22.99	3.57	11.23	3.69	3.99	18.46	0.208	-0.046	0.563	0.84	0.00	0 (0)

AFFM, appendicular fat free mass; DXA, dual energy X-ray absorptiometry; FFM, fat free mass; ICC, intraclass correlation coefficient; LOA, limits of individual agreement. Bias calculated as DXA data - Prediction equation value; 5% tolerance between DXA and prediction equations (Prediction equation/DXA from ≤0.95 to ≤1.05).
