# Supplementary material for: Performance of Bioelectrical Impedance and Anthropometric Predictive Equations for Estimation of Muscle Mass in Chronic Kidney Disease Patients
Source: Front Nutr. 2021 May 21;8:683393. doi: 10.3389/fnut.2021.683393 (PMC8177428; doi:10.3389/fnut.2021.683393)
Supplement: Supplementary file 5 [file Table_4.pdf]

TABLE S4. Agreement between DXA and prediction equations in kidney transplant patients sample stratified by sex

Body Composition Variable	Men												Women											
	DXA or Prediction Equation		Bland-Altman analysis				ICC analysis			Pearson correlation		5% Tolerance	DXA or Prediction Equation		Bland-Altman analysis				ICC analysis			Pearson correlation		5% Tolerance
			Bias (DXA-Prediction)		LOA		ICC	(95%CI)							Bias (DXA-Prediction)		LOA		ICC	(95%CI)				
	$\bar{x}$	SD	$\bar{x}$	SD	lower	upper	r	lower	upper	r	p	% (n)	$\bar{x}$	SD	$\bar{x}$	SD	lower	upper	r	lower	upper	r	p	% (n)
	Cross-sectional data n = 48												Cross-sectional data n = 33											
**AFFM$_{DXA}$ (kg)**	22.56	3.42											14.53	2.25										
**AFFM$_{Sergi}$ (kg)**	21.59	2.60	0.97	1.39	-1.75	3.69	0.854	0.594	0.935	0.93	0.00	54 (26)	15.09	1.90	-0.56	1.12	-2.75	1.29	0.829	0.635	0.918	0.87	0.00	39 (13)
**AFFM$_{Kyle}$ (kg)**	23.36	2.96	-0.79	1.34	-3.41	1.83	0.887	0.726	0.946	0.92	0.00	52 (25)	15.75	2.14	-1.03	1.12	-3.22	1.16	0.815	0.565	0.920	0.87	0.00	36 (12)
**AFFM$_{Macdonald}$ (kg)**	21.40	2.75	1.16	1.75	-2.27	4.59	0.788	0.497	0.900	0.86	0.00	44 (21)	13.17	1.77	1.36	1.38	-1.34	4.06	0.630	0.055	0.849	0.79	0.00	21 (7)
**FFM$_{DXA}$ (kg)**	47.53	6.68											33.21	5.22										
**FFM$_{TianHGS}$ (kg)**	49.62	5.38	-2.09	3.47	-8.89	4.71	0.792	0.504	0.897	0.85	0.00	48 (23)	35.38	5.01	-2.11	3.36	-8.69	4.47	0.731	0.504	0.875	0.79	0.00	24 (8)
**FFM$_{TianMAMC}$ (kg)**	52.52	5.53	-4.99	3.28	-11.42	1.43	0.645	-0.077	0.878	0.87	0.00	25 (12)	36.76	5.13	-3.55	3.32	-10.05	2.95	0.646	0.026	0.863	0.79	0.00	15 (5)
**FFM$_{NooriHGS}$ (kg)**	41.38	7.13	6.15	6.34	-6.27	18.57	0.417	-0.026	0.690	0.58	0.00	27 (13)	23.86	4.70	9.40	4.66	0.26	18.53	0.206	-0.082	0.546	0.57	0.00	6 (2)
**FFM$_{NooriMAMC}$ (kg)**	47.71	5.07	-0.18	3.45	-6.94	6.58	0.833	0.721	0.903	0.86	0.00	44 (21)	44.46	4.71	-11.25	3.32	-17.75	-4.74	0.219	-0.039	0.590	0.78	0.00	3 (1)
**FFM$_{Hume}$ (kg)**	52.24	5.30	-4.70	3.68	-11.91	2.51	0.626	-0.048	0.858	0.83	0.00	25 (12)	40.37	4.96	-7.16	3.71	-14.43	0.11	0.371	-0.091	0.728	0.73	0.00	0 (0)
**FFM$_{Janssen}$ (kg)**	29.44	3.55	18.08	4.40	9.45	26.70	0.099	-0.026	0.355	0.79	0.00	0 (0)	18.19	2.45	15.01	3.44	8.26	21.75	0.086	-0.029	0.368	0.84	0.00	0 (0)
**FFM$_{Lee}$ (kg)**	33.31	3.40	14.21	4.44	5.50	22.91	0.142	-0.042	0.446	0.80	0.00	0 (0)	22.88	2.85	10.33	3.52	3.43	17.22	0.162	-0.048	0.492	0.77	0.00	0 (0)

AFFM, appendicular fat free mass; DXA, dual energy X-ray absorptiometry; FFM, fat free mass; ICC, intraclass correlation coefficient; LOA, limits of individual agreement. Bias calculated as DXA data - Prediction equation value; 5% tolerance between DXA and prediction equations (Prediction equation/DXA from ≤ 0.95 to ≤ 1.05).
